# Identification and expression profile of the soil moisture and *Ralstonia solanacearum* response CYPome in ginger (*Zingiber officinale*)

**DOI:** 10.7717/peerj.11755

**Published:** 2021-08-02

**Authors:** Mengjun Huang, Haitao Xing, Zhexin Li, Honglei Li, Lin Wu, Yusong Jiang

**Affiliations:** 1 College of Pharmaceutical Science and Chinese Medicine, Southwest University, Chongqing, Chongqing, China; 2 Research Institute for Special Plants, Chongqing University of Arts and Sciences, Yongchuan, Chongqing, China; 3 Chongqing Key Laboratory of Economic Plant Biotechnology, Yongchuan, Chongqing, China

**Keywords:** *Ralstonia solanacearum*, CYPome, *Zingiber officinale*, Soil moisture, Expression profiles

## Abstract

**Background:**

Cytochrome P450s play crucial roles in various biosynthetic reactions. Ginger (*Zingiber officinale*), which is often threatened by *Ralstonia solanacearum*, is the most economically important crop in the family Zingiberaceae. Whether the cytochrome P450 complement (CYPome) significantly responds to this pathogen has remained unclear.

**Methods:**

Transcriptomic responses to *R. solanacearum* and soil moisture were analyzed in ginger, and expression profiles of the CYPome were determined based on transcriptome data.

**Results:**

A total of 821 P450 unigenes with ORFs ≥ 300 bp were identified. Forty percent soil moisture suppressed several key P450 unigenes involved in the biosynthesis of flavonoids, gingerols, and jasmonates, including unigenes encoding flavonoid 3′-hydroxylase, flavonoid 3′,5′-hydroxylase, steroid 22-alpha-hydroxylase, cytochrome P450 family 724 subfamily B polypeptide 1, and allene oxide synthase. Conversely, the expression of P450 unigenes involved in gibberellin biosynthesis and abscisic acid catabolism, encoding ent-kaurene oxidase and abscisic acid 8′-hydroxylase, respectively, were promoted by 40% soil moisture. Under *R. solanacearum* infection, the expression of P450 unigenes involved in the biosynthesis of the above secondary metabolites were changed, but divergent expression patterns were observed under different soil moisture treatments. High moisture repressed expression of genes involved in flavonoid, brassinosteroid, gingerol, and jasmonate biosynthesis, but promoted expression of genes involved in GA anabolism and ABA catabolism. These results suggest possible mechanisms for how high moisture causes elevated susceptibility to *R. solanacearum* infection.

## Introduction

Cytochrome P450 (CYP, P450) enzymes are a group of monooxygenases that compose one of the largest protein superfamilies in the biosphere. P450s can usually be classified into families and subfamilies based on sequence homology and phylogeny, including 127 families that represent around 1% of all protein-coding genes found in plants (Nelson & Werck-Reichhart, 2011; Nelson et al., 1996). P450 enzymes play crucial roles in the biosynthesis of plant secondary metabolites, including sterols, fatty acids, hormones, biosignaling substances, and antibiotics (Biazzi et al., 2015; Chapple, 1998; Saika et al., 2014; Schuler, 1996; Umemoto et al., 2016). Published reports indicate that biotic and/or abiotic stresses can significantly affect the expression of several P450 genes, and modifying the expression of P450 genes can also alter stress responses in plants (Bell-Lelong et al., 1997; Krasensky & Jonak, 2012; Narusaka et al., 2004; Whitbred & Schuler, 2000; Yan et al., 2016). Based on genomic sequencing efforts utilizing next-generation DNA sequencing technology, an increasing number of cytochrome P450 complements (CYPomes) have been revealed, including those of *Arabidopsis thaliana* (245), *Oryza sativa* (332), *Carica papaya* (142), *Morus notabilis* (174), *Vitis vinifera* (315), *Populus trichocarpa* (310), and *Physcomitrella patens* (71) (Ma et al., 2014; Nelson, 2006; Nelson et al., 2008; Nelson et al., 2004; Paquette, Bak & Feyereisen, 2000). Based on high-throughput RNA Sequencing (RNA-Seq), CYPomes can be also identified and characterized well for species without reference genome (Tao et al., 2017) .

Ginger (*Zingiber officinale* Roscoe), one of the most economically important species in the plant family *Zingiberaceae*, is used worldwide as a spice, a flavoring agent in the beverage, bakery, and confectionary industries, and an active ingredient in the pharmaceutical industry (Mahomoodally et al., 2021). China, India, and Indonesia are the major global ginger producers. The total Chinese ginger production reached 9.95 million tons in 2019, corresponding to 2.84  × 10^5^ hm^2^ (according to the Ministry of Agriculture and Rural Affairs of the PRC). As a crop that is asexually propagated, ginger is susceptible to a number of diseases (Jiang et al., 2018b; Prasath et al., 2014). Bacterial wilt is one of the most important production constraints in ginger cultivation (Prasath et al., 2014). This lethal wilting disease usually results in dysfunction of the vascular bundle system, which is responsible for the transportation of water, nutrients, and signaling molecules. *Ralstonia solanacearum*, one of the pathogenic bacteria that causes bacterial wilt, can spread through the xylem vessels and colonize and propagate within the xylem of ginger stems, ultimately causing infected plants to wilt and die (Denny & Baek, 1991; Peeters et al., 2013). While *R. solanacearum* has a wide host range and can infect over 300 plant species in 44 families, different *R. solanacearum* strains have different hosts. The strains of this pathogen can be divided into five physiologic races and five biochemical types. The pathogenic strains in China belong to physiologic race 1 and biochemical types II, III, and IV (Liu et al., 2005). Current knowledge of ginger wilt is primarily limited to empirical conclusions obtained from field planting experiments. Ginger plants have been reported to be more susceptible to bacterial wilt disease under high temperatures and high soil moisture levels (Jiang et al., 2018b; Liu et al., 2005; Tahat & Sijam, 2010). However, the CYPome responses to *R. solanacearum* infection and high soil moisture remain largely unexplored.

In previous studies, we confirmed that high soil moisture elevates susceptibility of ginger to *R.solanacearum* infection (Jiang et al., 2018b; Li et al., 2018). RNA-Seq results have demonstrated that a small number of genes are involved in the response to high soil moisture and infection by *R. solanacearum*, while a large number of genes are involved in defense against *R. solanacearum* infection (Jiang et al., 2018b). In this study, we first identified the CYPome of *Z. officinale*, and then characterized the expression patterns of these genes to soil moisture and *R. solanacearum* infection.

## Materials & Methods

### Plant materials

Tissue culture seedlings of *Z. officinale* Roscoe cv. Yujiang 1′(also known as ‘Southwest’ and bred in our laboratory in 2017) were stored in our laboratory. The following two media was optimal for adventitious bud induction and rapid proliferation of ginger plants: (1) MS with 6-BA (3 mg/L) and NAA (0.1 mg/L); (1) MS with 6-BA (5 mg/L), NAA (0.1 mg/L) and 0.2% activated carbon. The cultures were maintained at 25 °C under a light intensity of 3000 lux (14 h/d) for 90 d. Tissue culture seedlings with a height of ∼10 cm were transferred to 20 pots (six plants per pot) filled with steam sterilized nutrient soil, and the plants were grown in a cubicle greenhouse in which no other plants were planted (temperature, 25 °C; relative air humidity, 60%; photoperiod, 14 h of light at an intensity of 200 µEm^−2^s^−1^) for acclimation 30 days prior to experiments being conducted. The size of pots was 70  × 40  × 25 cm. The 20 pots were divided into five groups. Then, the water-filled pore space (WFPS) levels in pots were established at five increasing values, 10%, 20%, 25%, 30%, and 40%, with four pots (24 plants) for each WFPS condition. The soil moisture at depths of 0, 10, and 20 cm were measured twice a day with a soil moisture determinator (TZS-II, Zhejiang Top Cloud-agri Technology Co., Ltd., Hangzhou, China) and water was supplemented accordingly. This process continued for 30 days for the acclimation of plants to each WFPS condition. Next, prior to inoculation, the rhizomes of ginger plants in all pots were injured, and then inoculations were performed by drenching the soil in treatment groups using *R. solanacearum* suspension with a concentration of 10^6^ cfu/mL. Meanwhile, sterile water inoculation was used as the control. The *R. solanacearum* (race 1 biovar II) used in this study was isolated from a local ginger field without any genetic modification, and its specific host is ginger. The ginger growth was carefully inspected and recorded, tissue samples around the injured site of rhizomes were collected for each group. All equipment, materials, and facilities used in this study (including the cubicle greenhouse, pots, steam-sterilized nutrient soil, et al.), were strictly sterilized by ultraviolet irradiation or autoclaving before and after the experiment.

### RNA isolation, cDNA library preparation, and illumina sequencing

A total of four sample groups were collected three days after inoculation and immediately stored in liquid nitrogen for transcriptome sequencing, including LUN (low water-filled uninoculated, inoculation with sterile water under 10% WFPS), HUN (high water-filled uninoculated, inoculation with sterile water under 40% WFPS), LI (low water-filled inoculated, inoculation with *R. solanacearum* under 10% WFPS), and HI (high water-filled inoculated, inoculation with *R. solanacearum* under 40% WFPS), respectively. Each sample group had three biological replicates, and fresh rhizomes were collected from three different ginger plants and then pooled together as a biological replicate. Total RNAs were isolated from 12 samples using TRIzol^®^ reagent (Invitrogen, Carlsbad, CA, USA) according to the manufacturer’s instructions. Genomic DNAs were digested by DNase I (Fermentas, Burlington, ON, Canada). RNA quality and purity of samples were assessed by their optical density ratios (OD_260_/OD_230_ ratio and OD_260_/OD_280_ ratio, respectively) and RNA integrity number (RIN), which were measured by using the SMA3000 and the Agilent 2100 Bioanalyzer platforms, respectively. More than 10 µg of qualified total RNAs (RIN ≥ 7.0, 1.8 <OD_260_/OD_280_ <2.2, OD_260_/OD_230_ ≥2.0) of each biological replicate was then used for the following cDNA library construction according to the Illumina TruSeq (Illumina, San Diego, CA, USA) RNA library protocol. Transcriptome sequencing was conducted using an Illumina HiSeq 2500 platform (Illumina, San Diego, CA, USA) with 2 × 150 paired-end (PE) reads at BGI-Shenzhen, China.

### De Novo assembly, sequence annotation, and expression analyses

The raw reads generated by the HiSeq 2500 platform were filtered using Trimmomatic (v0.36) (Bolger, Lohse & Usadel, 2014), and submitted to the Sequence Read Archive (https://www.ncbi.nlm.nih.gov/sra) under the accession number PRJNA380972. Clean reads from four samples were pooled together and de novo assembled using Trinity (v2.3.1) (Grabherr et al., 2011). Unigenes longer than 300 bp were selected for the following analyses and submitted to the Transcriptome Shotgun Assembly (https://www.ncbi.nlm.nih.gov/genbank/tsa/, TSA) under the accession number PRJNA380972. Functional annotation was conducted using BlastP search (Camacho et al., 2009) against protein databases, including the NCBI non-redundant (NR), SwissProt, Eukaryotic Orthologous Groups of proteins (KOG), and Kyoto Encyclopedia of Genes and Genomes (KEGG) databases. Then, Gene Ontology (GO) annotations were retrieved via Blast2GO (Conesa & Gotz, 2008; Conesa et al., 2005). For each sample, clean reads were aligned back to the assembly separately using Bowtie2 (Langmead et al., 2009) to generate fragments per kilobase per million mapped fragments (FPKM) values. Differentially expressed genes (DEGs) were identified between each pair of sample type (i.e., LI-vs-LUN, HI-vs-HUN, HI-vs-LI, HUN-vs-LUN comparisons) by edgeR (the Empirical analysis of Digital Gene Expression in R) with thresholds of log_2_FC (log_2_fold-change) ≥ 1 and FDR <0.05 (Robinson, McCarthy & Smyth, 2010). Functional enrichment analyses were performed by a hypergeometric test with a threshold of *P*_adj_ ≤ 0.05.

### Identification and analyses of ZoP450s unigenes

Candidate P450 unigenes were first identified using cytochrome P450 as the search key word in the annotation of the de novo assembled transcriptomes. The candidates were then submitted to the NCBI database for protein Blast analyses to verify the annotation. P450 unigenes were then aligned using ClustalW (v1.83) (Thompson, Gibson & Higgins, 2002), and a maximum likelihood tree was constructed using MEGA6.0 (Tamura et al., 2013) and modified by iTOL (Letunic & Bork, 2016).

### qRT-PCR based expression validation

To validate the expression profiles obtained from RNA-Seq, 15 unigenes were randomly selected for primer design and real-time quantitative qRT-PCR analysis. The total RNA of four sequenced samples was treated with DNase I (Fermentas). First strand cDNA was then synthesized using oligo (dT) and random hexamers as primers using Moloney murine leukemia virus (M-MLV) reverse transcriptase (Invitrogen) according to the manufacturer’s instructions. Real-time PCR was performed using SYBR^®^ Premix Ex Taq™ II (TaKaRa, Shiga, Japan) on the IQ5 Real-Time PCR System (Bio-Rad, Hercules, CA, USA). The PCR was implemented under the following program: initial denaturation at 95 °C for 30 s, followed by 40 cycles of denaturation at 95 °C for 5 s, annealing at 60 °C for 30 s and extension at 72 °C for 30 s. Three biological replications were used and amplicons were used for melting curve analysis. The *Actin1* gene was selected as an internal control, using primer sequences 5′-TGGCATCTCTCAGCACATTCC-3′ and 5′-TGCACAATGGATGGGTCAGA-3′. Then the relative quantification results were calculated using the 2^−ΔΔ*CT*^ method (Livak & Schmittgen, 2001).

## Results

### Ginger CYPome identification and characterization

**Figure 1:**
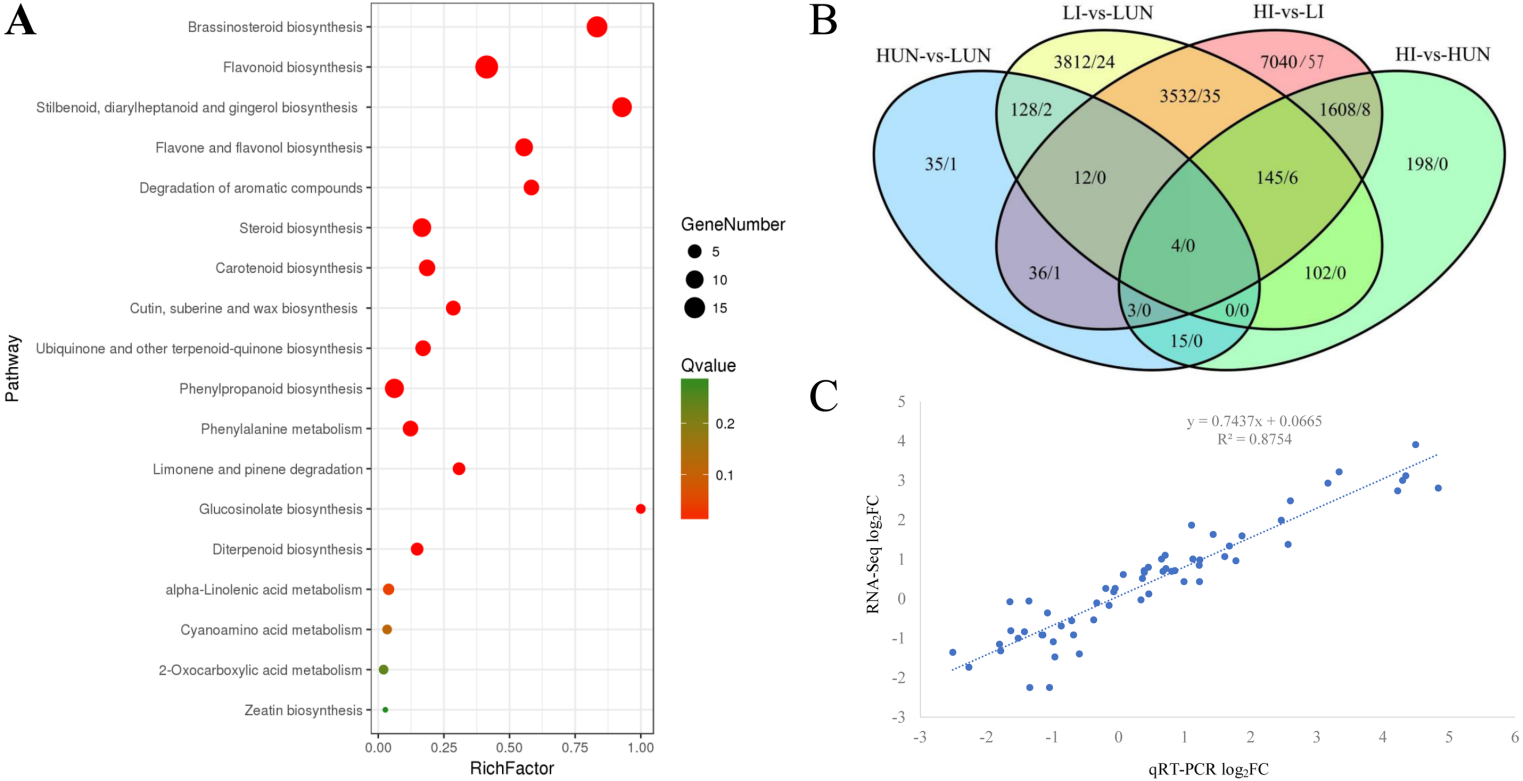
Kyoto Encyclopedia of Genes and Genomes (KEGG) Enrichment (A), differentially expressed unigenes (B) and expression validation (C) of the cytochrome P450 complement (CYPome). (A) Functional enrichment analyses of the CYPome were performed by a hypergeometric test with a threshold of *P*_adj_ ≤ 0.05 using the whole transcriptome as background. (B) DEGs were identified between each two samples by edgeR with thresholds of log_2_FC ≥ 1 and FDR < 0.05 (Robinson, McCarthy & Smyth, 2010). The number before each slash indicates the DEGs identified from the whole transcriptome, while the number after each slash indicates the DEGs identified from the CYPome. (C) the comparison of qRT-PCR and RNA-Seq quantitative results.

In our previous study, it was found that the growth of ginger showed significant difference under different soil moisture. With the increasing of WFPS, the tiller density and bacterial wilt symptoms elevated significantly (Jiang et al., 2018b). A comprehensive transcriptome of ginger (*Zingiber officinale*) with 369,905 unigenes was constructed previously using fresh rhizomes grown under different WFPS (Jiang et al., 2018b). In this study, based on keyword search and NCBI Blast verification, a total of 1063 unigenes were annotated as cytochrome P450 encoding genes, 821 of which possessed long ORFs (≥300 bp) and an average length of 1042.4 bp (Table S1). Among these 821 unigenes, 76 were grouped into the CYP71 family, 41 into CYP86, 24 into CYP51, 21 into CYP72, 21 into CYP94, and 18 into CYP75 (Table S1). Phylogenetic analyses clustered the 821 P450 unigenes into four groups (A, B, C, D), comprised of 321, 213, 162, and 125 members, respectively (Table S1). KEGG enrichment analyses demonstrated that the A group was enriched in steroid biosynthesis, brassinosteroid biosynthesis, retinol metabolism, and linoleic acid metabolism. The B group was enriched in flavonoid biosynthesis, flavone and flavonol biosynthesis, and cutin, suberine and wax biosynthesis. The C group was enriched in steroid biosynthesis, stilbenoid, diarylheptanoid and gingerol biosynthesis, and nitrogen metabolism. The D group was enriched in degradation of aromatic compounds, flavonoid biosynthesis, and retinol metabolism (Table S2). Gene Ontology (GO) annotation results demonstrated that these unigenes were mainly annotated for catalytic activity, binding, metabolic process, and single-organism process. Compared with the global transcriptome background, 821 P450 unigenes were mainly enriched (FDR < 0.05) in the brassinosteroid biosynthesis pathway (15 P450 unigenes), flavonoid biosynthesis pathway (19 P450 unigenes), and stilbenoid, diarylheptanoid and gingerol biosynthesis pathway (13 P450 unigenes) (Fig. 1A).

### Soil-moisture response

To globally profile the expression patterns of the transcriptome, differentially expressed unigenes were identified using edgeRwith thresholds of log_2_FC (log_2_ fold-change) ≥ 1 and FDR <0.05 (Robinson, McCarthy & Smyth, 2010). Only 233 DEGs were identified between the 10% (LUN) and 40% (HUN) soil moisture treatments, including 67 up-regulated and 166 down-regulated unigenes. Among the 821 P450 unigenes, only four were identified as DEGs, including Zoff283768, Zoff294948, Zoff057305, and Zoff304813 (Fig. 1B, Table S3). Zoff283768 encodes an alpha-humulene 10-hydroxylase (CYP71BA1, EC:1.14.13.150), which catalyzes the conversion of alpha-humulene to 8-hydroxy-alpha-humulene in zerumbone biosynthesis (Yu et al., 2011). The expression of Zoff283768 was significantly up-regulated by high soil moisture from 9.92 to 234.48 FPKM. Zoff294948 encodes flavonoid 3′-monooxygenase (CYP71A1, EC: 1.14.13.21), and its expression was down-regulated from 1309.61 (LUN) to 230.11 FPKM (HUN) by high moisture. Zoff057305 and Zoff304813 were expressed at low levels and significantly down-regulated by 40% soil moisture. Additionally, another alpha-humulene 10-hydroxylase (CYP71BA1) encoding sequence, Zoff291612, was also down-regulated by high soil moisture (24.38 versus 6.24 FPKM, log_2_FC = −1.97, *P* = 0.001, FDR > 0.05). Zoff449917 also encodes flavonoid 3′-monooxygenase (CYP71A1) and was down-regulated (138.31 versus 61.92 FPKM, log_2_FC = −1.16, *P* = 0.036, FDR >  0.05).

*Ralstonia solanacearum* inoculation, a total of 12,380 unigenes were differentially expressed under different soil moisture treatments (HI-vs-LI), including 107 P450 unigenes (Fig. 1B, Table S3). Seven of the 821 P450 unigenes were involved in ubiquinone and other terpenoid-quinone biosynthesis pathway, and six of them were identified as DEGs between the LI and HI groups (Zoff020741, Zoff020742, Zoff262685, Zoff262686, Zoff262690, Zoff262691). Additionally, the DEGs were significantly enriched in the phenylalanine metabolism pathway (7/10), stilbenoid, diarylheptanoid and gingerol biosynthesis pathway (7/13), phenylpropanoid biosynthesis pathway (6/12), flavonoid biosynthesis pathway (7/19), and degradation of aromatic compounds pathway (7/20). Most of these DEGs (7/10) were up-regulated by high soil moisture.

A comparison of the HUN-vs-LUN and HI-vs-LI DEGs revealed that Zoff283768 (a member of B group) was the only P450 unigene that was significantly differentially expressed between different soil moisture treatment regardless of *R. solanacearum* inoculation status (Fig. 1B). Its expression levels were 9.92, 234.48, 11.35, and 1145.36 FPKM in LUN, HUN, LI, and HI, respectively. Further analyses demonstrated that members of group B accounted for the largest proportion of the P450 DEGs. Among the 107 P450 DEGs identified between the LI and HI samples, 38, 28, 22, and 19 members belonged to groups B, A, C, and D, respectively, while such HUN-vs-LUN DEGs numbered 2, 1, 1, and 0, respectively.

### Response to *Ralstonia solanacearum*

The published literature has demonstrated that high soil moisture elevates susceptibility to *R. solanacearum* infection, resulting in ginger bacterial wilt (Jiang et al., 2018b). A total of 67 and 14 P450 unigenes were identified as DEGs in LI-vs-LUN and HI-vs-HUN comparisons, respectively (Table S3). Under high soil moisture, 12 of the 14 P450 DEGs were down-regulated by *R. solanacearum* infection, except Zoff018325 and Zoff262669. Conversely, 52 P450 DEGs identified under low soil moisture were up-regulated by *R. solanacearum* infection.

The identified P450 DEGs shared between the LI-vs-LUN and HI-vs-HUN comparisons were composed of six DEGs, including Zoff215340, Zoff251597, Zoff269538, Zoff221390, Zoff206943, and Zoff082186. Zoff215340 encodes a flavoprotein monooxygenase, and its expression was up-regulated by 6.93 times by *R. solanacearum* infection under low moisture, but down-regulated by 110.30 times by *R. solanacearum* infection under high moisture. Zoff251597 encodes an abscisic acid 8′-hydroxylase, and its expression was up-regulated by 5.60 times by *R. solanacearum* under low moisture, but down-regulated by 15.50 times by the pathogens under high moisture. Zoff269538 encodes an alkane hydroxylase MAH1-like protein. Infection with *R. solanacearum* reduced the expression of this unigene by 3.86 times and 25.76 times under low and high moisture, respectively. Zoff221390 encodes *o*-methylsterigmatocystin oxidoreductase, and *R. solanacearum* stimulated its expression (17.61 times) under low moisture but reduced its expression (28.65 times) under high moisture. Zoff206943 (CYP704C1) and Zoff082186 (CYP94C1) showed the same trend as Zoff215340, Zoff251597 and Zoff221390. Statistics showed that two of these six P450 DEGs (Zoff221390, Zoff251597) belong to group A, whereas the other four belong to group B. Further analyses found that members of group B account for the largest proportion of the identified P450 DEGs shared between the LI-vs-LUN and HI-vs-HUN comparisons. Among the 67 P450 DEGs identified between LUN and LI, 21, 20, 17, and 9 members belonged to groups B, A, C and D, respectively, while only 7, 4, 0, and 3 member in HI-vs-HUN DEGs.

To validate the RNA-Seq results, 15 P450 unigenes were randomly selected for qRT-PCR analysis (Table S4). The relative quantification results were calculated by the 2^−ΔΔ*CT*^ method (Livak & Schmittgen, 2001). Then, the log_2_FC were calculated and compared with that from RNA-Seq, yielding a correlation coefficient between the RNA-Seq and qRT-PCR data of *R*^2^ = 0.86 (Fig. 1C).

### Brassinolide response

**Figure 2:**
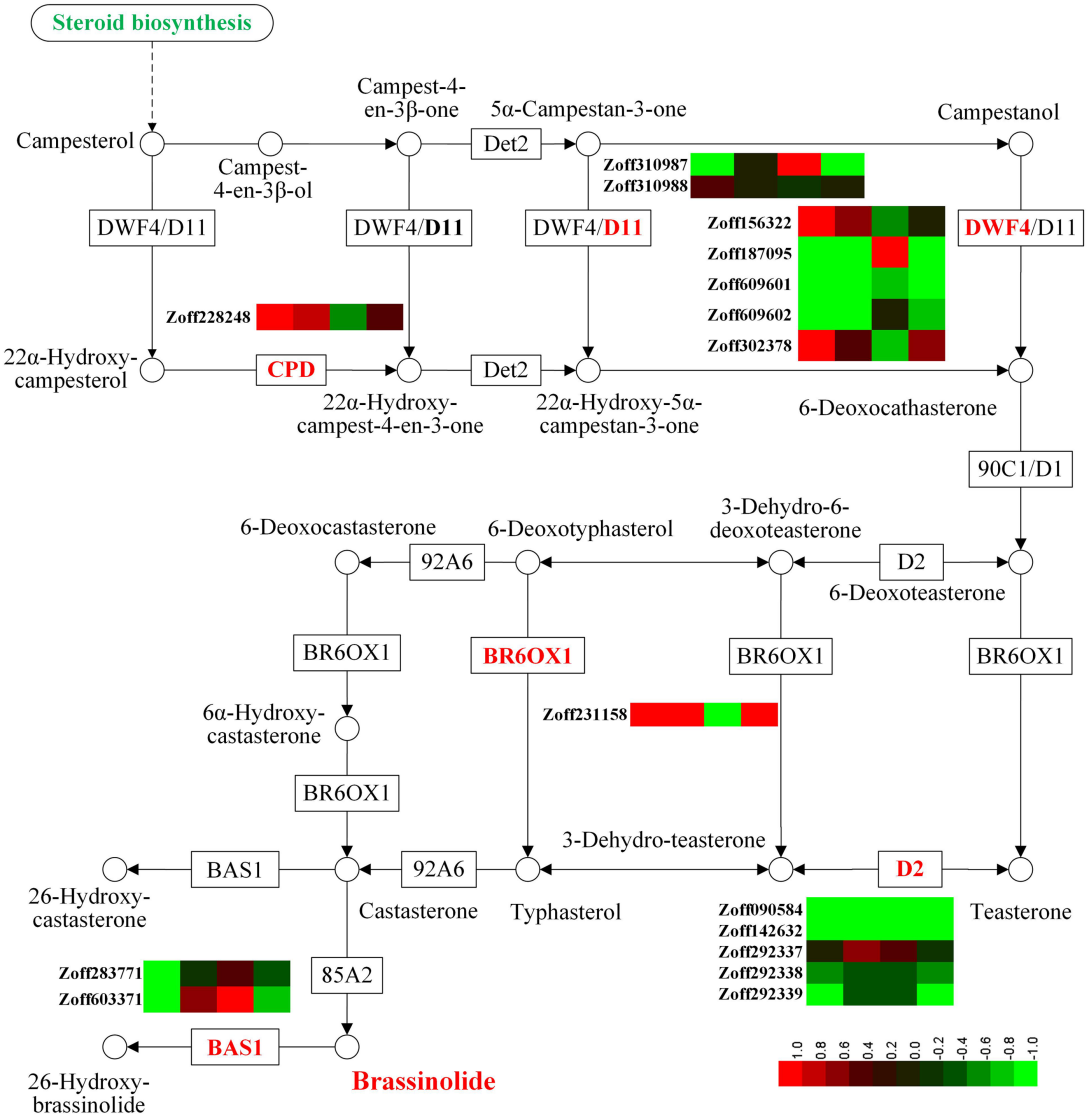
Expression of P450 unigenes involved in brassinolide biosynthesis. A heatmap was drawn according to log_2_FC values using HemI (v1.0) (Deng et al., 2014). log_2_FC was calculated pairwise based on the expression level for each P450 unigene. Columns from left to right correspond to log_2_(HI/LI), log_2_(HUN/LUN), log_2_(LI/LUN), and log_2_(HI/HUN) value, respectively.

Brassinosteroids regulate root growth, development, and symbiosis (Wei & Li, 2016). In this study, sixteen P450 unigenes involved in brassinosteroid biosynthesis were identified, including five steroid 22-alpha-hydroxylase unigenes (CYP90B1, DWF4, EC:1.14.13.-), two cytochrome P450 family 724 subfamily B polypeptide 1 unigenes (CYP724B1, D11, EC:1.14.13.-), one cytochrome P450 family 90 subfamily A polypeptide 1 unigenes (CYP90A1, CPD, EC:1.14.-.-), five steroid 3-oxidase unigenes (CYP90D2, D2, EC:1.14.-.-), one brassinosteroid-6-oxidase 1 unigene (CYP85A1, BR6OX1, EC:1.14.-.-), and two PHYB activation tagged suppressor 1 unigenes (CYP734A1, BAS1, EC:1.14.-.-) (Fig. 2, Table S5). Among the 16 P450 unigenes involved in brassinolide biosynthesis, 10 were group A members, while members of groups B, C, and D numbered 2, 3, and 1, respectively. In response to high moisture, the most highly expressed DWF4 unigene (Zoff187095) was down-regulated; its expression levels in the LI, HI, LUN, and HUN samples were 26.39, 0.50, 6.37, and 3.52 FPKM, respectively. The expression levels of the other three DWF4 unigenes, Zoff156322, Zoff609601, and Zoff609602, were much lower than those of Zoff187095. Zoff310987 encodes D11, and its expression levels were 25.11 and 5.47 FPKM in LI and HI samples, respectively. Conversely, the CPD (Zoff228248) and BR6OX1 (Zoff231158) encoding unigenes were both up-regulated by high moisture. Under *R. solanacearum* infection, the expression level of CPD (Zoff228248) was up-regulated from 14.83 FPKM in LI to 59.87 FPKM in HI, and up-regulated from 19.93 FPKM in LUN to 39.59 FPKM in HUN without *R. solanacearum* infection. The expression of BR6OX1 (Zoff231158) was up-regulated from 6.81 FPKM in LI to 134.06 FPKM in HI, and 11.89 FPKM in LUN to 27.89 FPKM in HUN.

In response to *R. solanacearum* infection, the most highly expressed DWF4 (Zoff187095) was up-regulated under low moisture, but was down-regulated under high moisture. The most highly expressed D11 (Zoff310987) and BSA1 (Zoff283771) showed the same expression pattern as Zoff187095 in response to *R. solanacearum*, but CPD (Zoff228248) and BR6OX1 (Zoff231158) demonstrated an opposite trend.

### Flavonoid response

**Figure 3:**
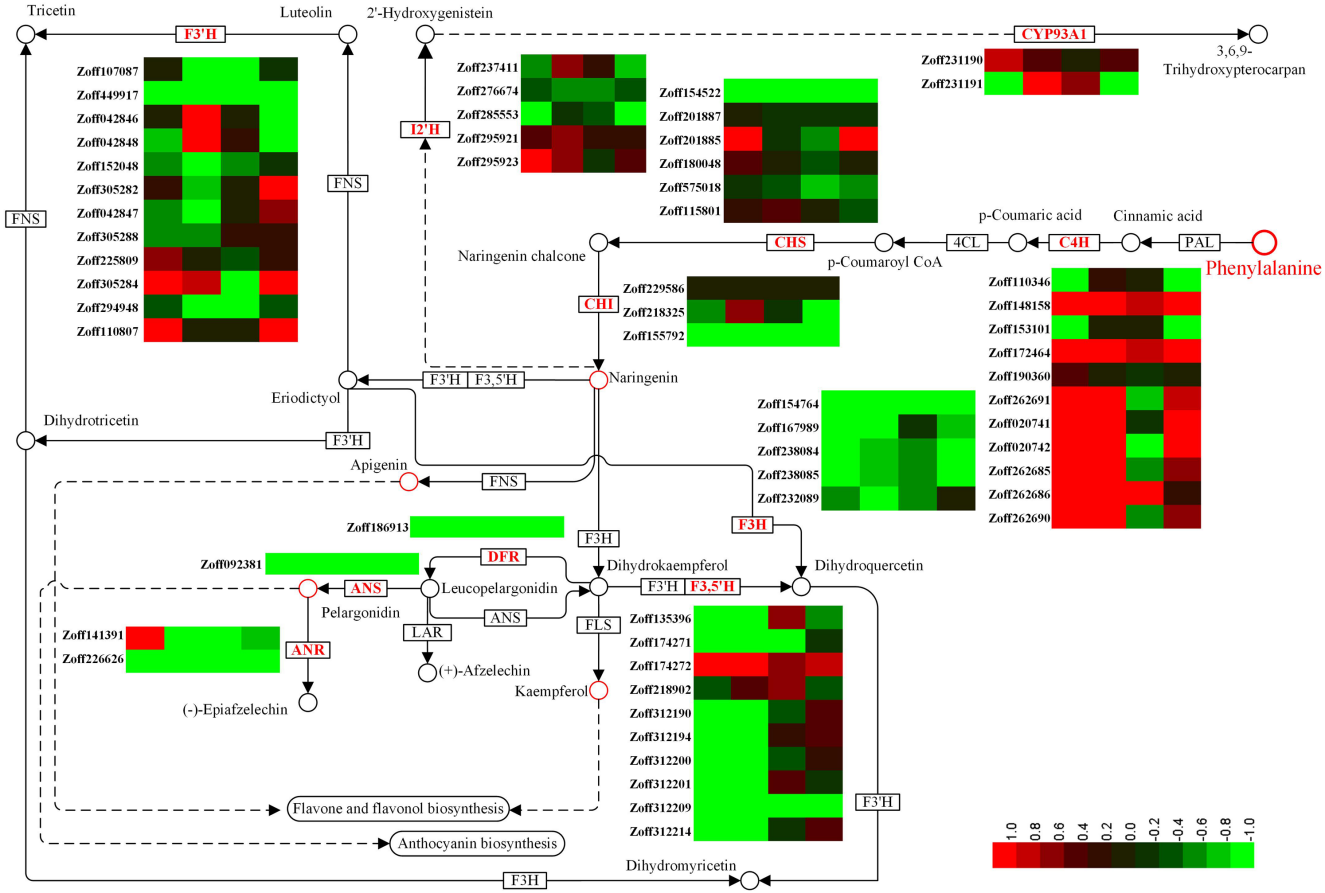
Expression of P450 unigenes involved in flavonoid biosynthesis. Heatmap was drawn according to log_2_FC values using HemI (v1.0) (Deng et al., 2014). log_2_FC was calculated pairwise based on the expression level for each P450 unigene. Columns from left to right correspond to log_2_(HI/LI), log_2_(HUN/LUN), log_2_(LI/LUN), and log_2_(HI/HUN) value, respectively.

Flavonoids constitute a vast class of secondary metabolites, encompassing more than 10,000 structures (Harborne & Williams, 2000). Different flavonoids play various roles in plants by regulating several developmental processes (Brown et al., 2001; Buer & Djordjevic, 2009; Martin & Gerats 1993; Koes et al., 1990; Taylor & Grotewold, 2005). In this study, a total of 12 cinnamate 4-hydroxylase unigenes (C4H, CYP73A4, EC:1.14.13.11), 12 flavonoid 3′-hydroxylase unigenes (F3′H, CYP75B, EC:1.14.14.82), 10 flavonoid 3′,5′-hydroxylase unigenes (F3′5′H, CYP75A, EC:1.14.13.88), 5 isoflavone 2′-hydroxylase unigenes (I2′H, CYP81E1/E7, EC:1.14.13.89; 1.14.13.53), and 2 3,9-dihydroxypterocarpan 6a-monooxygenase unigenes (CYP93A1, EC:1.14.13.28) were identified. C4H is one of the universal key factors involved in lignin and flavonoid synthesis (Hahlbrock & Grisebach, 1979). Most C4H unigenes were up-regulated by high moisture. Zoff262691, one of the most highly expressed C4H unigenes, was up-regulated from 7.59 (LI) and 12.37 (LUN) to 216.82 (HI) and 111.20 FPKM (HUN) under high moisture, respectively. Another highly expressed C4H unigene, Zoff190360, had expression levels of 213.41, 243.25, 293.93, and 265.88 FPKM in the LI, LUN, HI, and HUN samples, respectively. The other four C4H unigenes, Zoff172464, Zoff148158, Zoff262685, and Zoff262690, also showed the same trend as Zoff262691. Under high moisture, Zoff148158, Zoff172464, Zoff190360, Zoff262691, Zoff020741, Zoff020742, Zoff262685, and Zoff262690 were up-regulated by *R. solanacearum* infection. Zoff148158, Zoff153101, and Zoff172464 were also up-regulated by the pathogens under low moisture, while the most highly expressed C4H unigene (Zoff190360) was slightly down-regulated (243.25 to 213.41 FPKM). F3′H is a key enzyme involved in flavonoid biosynthesis (Deshmukh et al., 2018; Forkmann, Heller & Grisebach, 1980). The expression levels of most F3′H unigenes were suppressed by high moisture (Fig. 3, Table S5). For example, expression of Zoff294948 was down-regulated from 221.23 in LI to 175.87 FPKM in HI, and from 1039.61 in LUN to 230.11 FPKM in HUN. Infection with *R. solanacearum* suppressed the expression of Zoff449917, Zoff225809, and Zoff294948 under low moisture, and suppressed Zoff449917 and Zoff294948 under high moisture. High moisture down-regulated almost all of the F3′5′H encoding unigenes, whereas there were no significant expression changes observed under *R. solanacearum* infection. I2′H catalyses the hydroxylation of isoflavones, daidzein, and formononetin. In this study, five I2′H encoding unigenes were identified. However, no consistent change among these unigenes was observed. CYP93A1 is another key enzyme involved in flavonoid biosynthesis. Zoff231190 and Zoff231191 were both annotated as encoding CYP93A1. Zoff231191 had low expression across four samples, while the expression levels of Zoff231190 were 14.62, 26.82, 12.97, and 19.15 FPKM in the LI, HI, LUN and HUN samples, respectively. These results showed that the expression of Zoff231190 was up-regulated by high moisture (Fig. 3).

### Gibberellin and Jasmonate response

**Figure 4:**
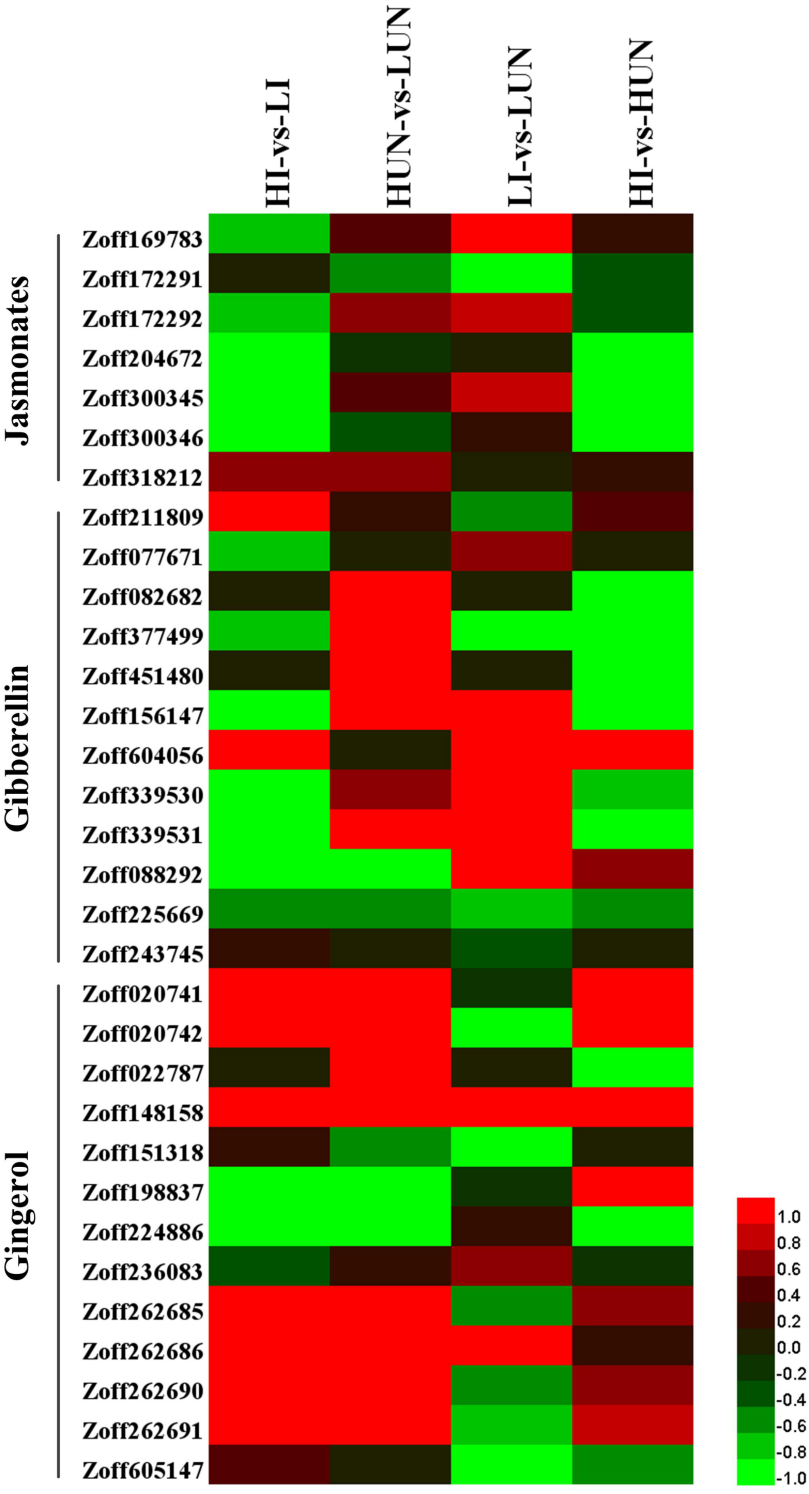
Expression of P450 unigenes involved in gibberellin, jasmonate, and gingerols biosynthesis. Heatmap was drawn according to log_2_FC values using HemI (v1.0) (Deng et al., 2014). log_2_FC was calculated pairwise based on the expression level for each P450 unigene. Columns from left to right correspond to log_2_(HI/LI), log_2_(HUN/LUN), log_2_(LI/LUN), and log_2_(HI/HUN) value, respectively.

Gibberellins (GAs) are an important class of plant growth regulators. Ent-kaurene oxidase (KO, CYP701A, EC:1.14.13.78), ent-kaurenoic acid oxidase (KAO1, CYP88A, EC:1.14.13.78), cytochrome P450 monooxygenase 1 (GA14 synthase, P450I, CYP68A), cytochrome P450 monooxygenase 2 (P450II, CYP68B), and cytochrome P450 monooxygenase 3 (P450III, CYP69A) are the P450 proteins involved in gibberellin biosynthesis. In this study, nine KO encoding unigenes (Zoff211809, Zoff077671, Zoff082682, Zoff377499, Zoff451480, Zoff156147, Zoff604056, Zoff339530, Zoff339531) were identified (Fig. 4, Table S5). The average expression levels of Zoff211809 were 47.87, 106.94, 64.16, and 77.59 FPKM in LI, HI, LUN, and HUN samples, respectively. Whereas the other KO encoding unigenes were all expressed at low levels in different samples. Additionally, three KAO encoding unigenes (Zoff088292, Zoff225669, Zoff243745) were identified in this study (Fig. 4, Table S5). Expression levels of the most highly expressed unigene, Zoff225669, were 12.91, 9.46, 21.79, and 14.45 FPKM in LI, HI, LUN, and HUN samples, respectively.

Allene oxide synthase (AOS, CYP74) is the first gene in the jasmonate (JA) biosynthetic pathway. A total of seven AOS encoding unigenes were successfully identified (Zoff169783, Zoff172291, Zoff172292, Zoff204672, Zoff300345, Zoff300346, and Zoff318212) (Fig. 4, Table S5). High soil moisture suppressed the expression of Zoff169783, Zoff204672, Zoff300345, and Zoff300346 when ginger plants were inoculated with *R. solanacearum*. Zoff300345 and Zoff300346 were the two most highly expressed AOS unigenes, and the expression of these two unigenes were 225.64 and 233.16 FPKM in LI samples, respectively, but decreased to 64.69 and 82.86 FPKM, respectively, in HI samples. However, high moisture slightly up-regulated several AOS unigenes, including Zoff169783, Zoff300345, and Zoff318212, when ginger was uninfected with *R. solanacearum*. Comparison of the expression of these AOS unigenes between LI and LUN, and between HI and HUN revealed that *R. solanacearum* infection up-regulated the expression of Zoff300345, Zoff318212, Zoff169783, and Zoff172292 under low moisture, but down-regulated the two most highly expressed AOS unigenes, Zoff204672 and Zoff300345, under high moisture.

### Gingerol biosynthesis

Among the 821 P450 unigenes, a total of 13 unigenes were involved in stilbenoid, diarylheptanoid, and gingerol biosynthesis (Fig. 4, Table S5). In response to high moisture, six highly expressed unigenes showed obvious differences among samples. For example, the highly expressed unigenes Zoff198837 and Zoff224886 were suppressed. The expression levels of Zoff198837 were 12.03, 5.33, 12.62, and 2.42 FPKM, and those for Zoff224886 were 68.51, 1.67, 56.27, and 22.87 FPKM in LI, HI, LUN, and HUN, respectively. The expression levels of Zoff148158, Zoff262685, Zoff262690, and Zoff262691 were up-regulated. Further analyses found that the four up-regulated unigenes all encode C4H, one of the universal key factors involved in lignin and flavonoid synthesis branches of phenylalanine metabolism networks (Hahlbrock & Grisebach, 1979). Based on the down-regulated expression of these two unigenes, high moisture suppressed gingerols biosynthesis.

## Discussion

Cytochrome P450s comprise one of the largest protein superfamilies in the biosphere. P450s can usually be classified into 127 families that represent around 1% of the protein-coding genes in plants (Nelson & Werck-Reichhart, 2011; Nelson et al., 1996). However, the number of CYP genes identified in each species varies considerably. Benefitting from the recent genome sequencing efforts, the identification of P450 genes has become practical in many species (Nelson, 2006; Nelson et al., 2008; Nelson et al., 2004; Paquette, Bak & Feyereisen, 2000). In this study, a total of 821 P450 unigenes (ORFs ≥ 300 bp) were identified, which was much more than in the other plant species. Although there must be many redundant unigenes, this data provides a preliminary but global insight into the CYPome of this economically valuable plant. Published studies have reported that ginger plants are more susceptible to bacterial wilt disease under high temperature and high soil moisture (Jiang et al., 2018b; Liu et al., 2005; Tahat & Sijam, 2010). Without *R. solanacearum* infection, only 233 DEGs (log_2_FC ≥ 1, FDR <0.05) were identified between 10% (LUN) and 40% (HUN) soil moisture samples, and only four P450 unigenes were identified as DEGs. These results suggest that relatively small changes were induced by the 40% soil moisture treatment without *R. solanacearum* infection. Two of the four P450 DEGs were involved in zerumbone biosynthesis (Zoff283768) and flavonoid biosynthesis (Zoff294948) (Table 1). Zoff283768 was up-regulated, while Zoff294948 was significantly down-regulated. Another two P450 DEGs were also down-regulated by high soil moisture. Under *R. solanacearum* inoculation, a total of 12,380 unigenes and 107 P450 unigenes were identified as DEGs under different soil moisture treatments (HI-vs-LI). Functional enrichment analysis demonstrated that the P450 DEGs were enriched in the phenylalanine metabolism pathway (7/10) and flavonoid biosynthesis pathway (7/19), among others. In consideration of the function of flavonoids in stress response (Brunetti et al., 2013; Shirley, 1996), different expression levels of key genes involved in this pathway may affect resistance to bacterial wilt disease. Accordingly, we carefully analyzed the expression patterns of P450 unigenes involved in flavonoid biosynthesis. F3′H and F3′5′H were key P450 enzymes involved in this pathway (Deshmukh et al., 2018; Forkmann, Heller & Grisebach, 1980; Hahlbrock & Grisebach, 1979). Results demonstrate that the expression of most F3′H- and F3′5′H-encoding unigenes were suppressed by high moisture treatment, which suggests that high soil moisture suppressed the biosynthesis of some flavonoids, thus resulting in an increased susceptibility to bacterial wilt disease. As a precaution, we also analyzed other key genes involved in phenylalanine metabolism, and found that most C4H unigenes were up-regulated by high moisture. The other two universal key factors involved in flavonoid and lignin biosynthesis branches, PAL and 4CL (Hahlbrock & Grisebach, 1979), were also found to be mostly up-regulated by high moisture. Additionally, most (highly expressed) lignin biosynthesis involved unigenes, such as coumarate 3-hydroxylase (C3H) and cinnamoyl-CoA reductase (CCR) encoding unigenes, were up-regulated by high moisture (see the supplementary data for our previous study (Jiang et al., 2018b). Flavanone 3-hydroxylase (EC: 1.14.11.9, F3H), anthocyanidin reductase (EC:1.3.1.77, ANR), anthocyanidin synthase (EC:1.14.20.4, ANS) and dihydroflavonol 4-reductase (EC: 1.1.1.234, DFR) are several other key enzymes (not P450) involved in flavonoid biosynthesis (Hahlbrock & Grisebach, 1979). In response to high soil moisture, highly expressed unigenes encoding F3H (such as Zoff154764, Zoff232089, Zoff238084, Zoff238085), ANR (Zoff226626), ANS (Zoff092381) and DFR (Zoff186913) were down-regulated by the 40% soil moisture treatment (Fig. 3). These results suggest that high soil moisture suppressed the biosynthesis of flavonoids but promoted lignin biosynthesis and that the increased metabolic flux was directed into the lignin branch, which may be an important factor in the increased susceptibility to bacterial wilt disease.

**Table 1:** Expression abundances of some important P450 unigenes.

**SeqID**	**LUN**	**LI**	**HUN**	**HI**	**SeqID**	**LUN**	**LI**	**HUN**	**HI**
Zoff026559	0	0.03	0	0	Zoff251597	80.69	452.19	402.73	26
Zoff031249	0	0.27	0.1	0.07	Zoff268607	32.51	7.34	24.07	48.06
Zoff092381	13.43	3.74	0.85	0.19	Zoff268608	3.77	1.09	2.81	6.16
Zoff117424	0.03	7.22	0.39	0	Zoff269701	25.81	21.34	23.45	21.76
Zoff154764	14.49	4.76	4.36	2.41	Zoff279532	7.89	11.59	1.81	2.84
Zoff161215	13.47	81.63	14.99	5.3	Zoff283768	9.92	11.35	234.48	1145.36
Zoff186913	4.06	1.02	1.12	0.3	Zoff283771	39.94	54.99	38.69	29.68
Zoff187095	6.37	26.39	3.52	0.5	Zoff292829	25.23	21.54	20.77	16.37
Zoff204672	214.37	225.64	206.72	64.69	Zoff292832	15.56	15	12.87	12.86
Zoff211809	64.16	47.87	77.59	106.94	Zoff294948	1309.61	221.23	230.11	175.87
Zoff216312	116.72	295.94	149.94	95.52	Zoff300345	132.54	233.16	180.4	82.86
Zoff226553	12.82	11.19	8.52	6.77	Zoff310987	9.79	25.11	10.45	5.47
Zoff226626	4.36	1.16	0.39	0.13	Zoff310988	10.54	9.43	12.09	13.46
Zoff228248	19.93	14.83	39.59	59.87	Zoff318212	112.47	123.06	169.01	211
Zoff232089	20.93	14.32	10.21	10.31	Zoff374704	0.03	0	0	0.05
Zoff238084	80.35	56.07	46.52	3.64	Zoff449917	138.31	63.48	61.92	20.33
Zoff238085	250.21	188.29	146.08	18.3	Zoff465097	0	0.04	0.02	0.07
Zoff245593	57.96	126.91	50.9	12.52	Zoff465823	0.02	0.12	0.03	0.02
Zoff251591	4.06	10.84	14.63	14.9					

As an important medicinal component of ginger, gingerol exhibits many biological properties, including anti-cancer, anti-microbial, antioxidant, and anti-inflammatory properties (Fan, Yang & Bi, 2015; Roufogalis, 2014). These components are the major pungent compounds present in the rhizomes of ginger, and are a class of metabolite of the lignin branch in the phenylalanine metabolism pathway (Jiang et al., 2018a). Under the 40% soil moisture treatment, down-regulation of the highly expressed P450 unigenes involved in gingerol biosynthesis suggested that high moisture also suppressed the synthesis of these pungent compounds.

Plant hormones, including ABA, GA, BR, jasmonic acid (JA), salicylic acid (SA), and ethylene (ET), develop a signaling crosstalk network regulating stress responses (Ku et al., 2018; Kundu & Gantait, 2017; Sun, 2011). Accompanied by changes in the growing environment, plants adjust their metabolism of these phytohormones. Brassinosteroids (BRs) are a group of steroidal compounds that can protect plants from environmental stresses (Krishna, 2003; Nakashita et al., 2003). As of the present, more than 70 BRs have been isolated in plants(Lv & Li, 2020). DWF4 and CPD are both rate-limiting enzymes in the BR biosynthesis pathway (Liu et al., 2007; Zhou et al., 2016), and down-regulation of DWF4 and D11, and up-regulation of CPD (Zoff228248) and BR6OX1 unigenes may indicate the suppression of BR biosynthesis by 40% soil moisture. As BRs can confer resistance in diverse crops to a broad array of pathogens (De Bruyne, Höfte & De Vleesschauwer, 2014; Szekeres et al., 1996) [22,178], the suppression of BRs could contribute to susceptibility to *R. solanacearum* infection. Additionally, the first JA biosynthetic involved gene (AOS, CYP74) was suppressed by 40% soil moisture, which may also result in an elevated susceptibility to *R. solanacearum* infection when ginger plants are inoculated with pathogens, because JA positively regulates defense responses against phytopathogens (Jiang et al., 2015; Zhang et al., 2017). GAs are negative regulators of disease resistance in plants (Qin et al., 2013; Yang et al., 2008), and thus, the up-regulation of unigenes involved in GAs biosynthesis, such as KO-encoding unigene (Zoff211809), may negatively affect resistance to *R. solanacearum*.

Generally, endogenous abscisic acid (ABA) content changes dynamically in plants responding to environmental conditions (Zeevaart, 1980). In vegetative tissues, ABA mediates adaptive responses to abiotic and biotic stresses, including drought, cold, high salinity, heat, and pathogen infection (De Torres-Zabala et al., 2007; Robertson et al., 1994; Yamaguchi-Shinozaki & Shinozaki, 2006; Zhu, 2002). ABA 8′-hydroxylase is a key enzyme in ABA catabolism and is encoded by CYP707A genes (Okamoto et al., 2009). The expression levels of CYP707A1 and CYP707A3 were previously shown to increase when wild-type *A. thaliana* plants were transferred to high-humidity conditions (relative humidity 90%), followed by a reduction of ABA levels (Okamoto et al., 2009). Zoff251597 encodes an abscisic acid 8′-hydroxylase, and the expression of this unigene was significantly up-regulated from 80.69 in the LUN to 402.73 FPKM in the HUN treatment, which likely resulted in the reduction of ABA levels and greater susceptibility to bacterial wilt disease. Additionally, Zoff026559, Zoff161215, Zoff216312, Zoff251591, Zoff465823, Zoff374704, Zoff031249, and Zoff465097 also encode ABA 8′-hydroxylase. Zoff216312, Zoff161215, and Zoff251591 showed the same expression patterns as observed in Zoff251597 (FDR > 0.05), while the other ABA 8′-hydroxylase unigenes were expressed at extremely low levels. Further analyses of ABA biosynthesis related genes, including NCED (9-cis-epoxycarotenoid dioxygenase) and ZEP (Zeaxanthin epoxidase) unigenes, showed that Zoff245593, Zoff279532, Zoff117424, Zoff226553, Zoff268607, Zoff269701, Zoff268608, Zoff292829, and Zoff292832 were more or less down-regulated in HUN samples when compared with LUN samples. These results indicate that high soil moisture suppressed ABA biosynthesis and promoted the degradation of this growth regulator.

Ginger is susceptible to a number of diseases. Among these, bacterial wilt disease caused by *R. solanacearum* is one of the most critical production constraints to ginger cultivation (Jiang et al., 2018b; Prasath et al., 2014). Bacterial wilt disease induces a dysfunction of the vascular bundle system, which is responsible for the transportation of water, nutrients, and signaling molecules within plants. Field cultivation experiments have suggested that high soil moisture increases ginger susceptibility to *R. solanacearum* infection. The down-regulated expression of the xyloglucan endotransglucosylase gene (*XET*) and hypersensitive response related gene (*PRX* and *CYP*) indicated that the normal cell wall metabolism may be disturbed and that the hypersensitive response may be inhibited by *R. solanacearum* infection (Jiang et al., 2018b). In this study, most P450 DEGs (12/14) were down-regulated when ginger plants were cultivated under high soil moisture conditions and infected with *R. solanacearum* (HI-vs-HUN), but most P450 DEGs (52/67) were up-regulated under low moisture (LI-vs-LUN). The most highly expressed DWF4 unigene (Zoff187095) was up-regulated under low moisture but down-regulated under high moisture when plants were infected. The highly expressed unigenes encoding D11, BSA1, F3 ′H, and AOS showed the same expression pattern as Zoff187095 (DWF4) in response to *R. solanacearum*. These results suggest that ginger plants have different *R. solanacearum* responsive CYPomes under different soil moisture conditions. Under *R. solanacearum* infection, ginger exhibited biosynthesis of multiple secondary metabolites, including those of brassinolides, jasmonates, and flavonoids.

## Conclusions

CYPome analysis based on next-generation DNA sequencing identified 821 P450 unigenes (with ORFs ≥ 300 bp). Expression profiling of the CYPome indicated that high soil moisture suppressed the biosynthesis of flavonoids, gingerols, jasmonates, and abscisic acid, but promoted the biosynthesis of gibberellins, thus likely resulting in elevated susceptibility to *R. solanacearum* infection. This study provides preliminary but broad insights into the cause of bacterial wilt disease in ginger, provides a theoretical basis for soil moisture control in ginger cultivation and development of genetic resources for ginger breeding.

##  Supplemental Information

10.7717/peerj.11755/supp-1 Supplemental Information 1 Annotation and Expression Levels of P450 Unigenes

10.7717/peerj.11755/supp-2 Supplemental Information 2 Kyoto Encyclopedia Genes and Genomes (KEGG) Enrichment Analyses of Different P450 Unigene Groups

10.7717/peerj.11755/supp-3 Supplemental Information 3 Differentially Expressed Unigenes Identified Between Each Two Samples P450 DEGs were marked in bold.

10.7717/peerj.11755/supp-4 Supplemental Information 4 Expression validation using qRT-PCR

10.7717/peerj.11755/supp-5 Supplemental Information 5 Expression Profiling of P450 Unigenes Involved in Brassinolide, Flavonoid, Jasmonate, Gibberellin, and Gingerol Biosynthesis

## Additional Information and Declarations

### Data Availability

The following information was supplied regarding data availability: The clean reads are available at the Sequence Read Archive: PRJNA380972.
